# Usage of contraception among the married women in India, 2019–21: a cross-sectional study

**DOI:** 10.1186/s40834-025-00375-2

**Published:** 2025-09-30

**Authors:** Chander Shekhar, Harihar Sahoo, Labhita Das

**Affiliations:** 1 International Institute for Population Sciences, Mumbai, India; 2 Department of Family & Generations, International Institute for Population Sciences, Mumbai, Maharashtra 400088 India; 3 Department of Community Medicine, Parul Institute of Medical Sciences & Research (PIMSR), Parul University, Vadodara, Gujarat India

**Keywords:** Contraceptive methods, Cross-sectional, India, NFHS, Unmet need

## Abstract

**Background:**

Contraceptive use plays a significant role in reducing fertility and improving maternal and child health. Understanding utilization patterns and preferences is essential for addressing unmet needs in family planning among married women in India.

**Objective:**

This study aims to analyse the usage of contraceptive methods among the married women aged 15–49 years in India, and to identify the factors associated with contraceptive uptake.

**Methods:**

Cross-sectional data from the fifth round of the National Family Health Survey, (2019–21) were used for this analysis. NFHS is a periodic cross-sectional survey conducted using a multistage household sampling design in India. The total sample consisted of 512,408 women aged 15–49 years currently in a marital union. Cross-tabulation was conducted to evaluate the levels of contraceptive use, and logistic regression was utilized to identify factors associated with contraceptive uptake. The entire analysis was performed using the STATA 17.0 statistical software.

**Results:**

Contraceptive usage among married women has increased from 53.5% in NFHS-4 (2015–16) to 65.7% in NFHS-5 (2019–21). The likelihood of contraception usage among the married women in India is significantly influenced by the factors like age of the women, educational attainment, desire for children, caste, religion, wealth index, exposure to mass media and place of residence.

**Conclusion:**

Despite advancements in reproductive health, contraceptive uptake among married women in India remains insufficient. To address this, interventions should prioritize the provision of diverse contraceptive options, along with comprehensive counselling, to emphasize the benefits and proper use of these methods. Addressing socio-demographic disparities is essential for improving equitable access to family planning services.

## Introduction

Globally, the concept of unmet need for contraception highlights the gap between women who wish to avoid or delay pregnancies and those who actually use contraceptive methods. Initially introduced as the "KAP-Gap" in the 1970 s, this concept became a cornerstone of family planning advocacy [1]. Contraception refers to the deliberate prevention of pregnancy through various methods, including behavioural, chemical, medical, or surgical techniques, and is pivotal in advancing both personal and public health goals [2]. Its benefits extend beyond averting unintended pregnancies, contributing to improved maternal and child health and supporting population management efforts [3]. Modern contraceptive methods such as sterilization, intrauterine devices (IUDs), implants, injections, oral contraceptives, and spermicides offer significantly higher efficacy compared to traditional methods like withdrawal or rhythm techniques [4]. However, the adoption of modern contraceptives remains low in many regions due to a combination of socioeconomic constraints, cultural opposition, and widespread misconceptions about their side effects or long-term health impacts [5, 6].

Across Asia, access to and use of contraception exhibit substantial variation, shaped by cultural, religious, and economic factors [5]. In South Asia, for instance, unmet needs for contraception remain significant despite a rising demand for family planning services [7]. Contraceptive practices are heavily influenced by gender norms and religious beliefs, with Muslim women using contraceptives less frequently than non-Muslims [3]. Additionally, limited awareness, fear of adverse effects, and resistance from male partners pose further obstacles to the adoption of effective methods. Studies indicate that involving men in family planning discussions can lead to improved reproductive health outcomes by reducing unintended pregnancies and fostering joint decision-making within families [8].

India exemplifies the complex challenges associated with the adoption of contraception [6].While the country has made considerable progress in enhancing family planning services, persistent hurdles such as misinformation and adolescent misconceptions about contraceptive methods continue to impede usage [9]. In 1952, India launched its family planning program as a cornerstone of its national population policy, aimed at curbing rapid population growth and reducing poverty [10, 11]. Despite being among the pioneering developing nations to prioritize family planning as a significant objective of central government policy, India's population has more than doubled since 1961 [10]. Contraceptive use remains a subject of ongoing scientific interest due to its role in reducing fertility rates and enhancing maternal and child health outcomes in India [12]. However, there exists considerable diversity in contraceptive prevalence and demand for family planning across different Indian states [12]. Until the mid-1990s, reproductive and child health programs in India primarily targeted women exclusively, men were excluded from the programs [13]. In 1998, the country adopted an informed choice model of service delivery, which remains in practice today [11].

The positive impact of increased contraceptive use in India is undeniable. Wider adoption could significantly reduce maternal mortality, lengthen intervals between births, and enhance maternal and child health outcomes [14]. Limited choices and access to family planning services, poor quality of available services, cultural and religious opposition, fear of adverse effects, and gender-based barriers contribute to the high rate of unmet need for contraception in countries like India [15]. Traditional methods which are considered to be the less effective methods are still prevalent in several communities, influenced by cultural norms and societal expectations [16]. Female sterilization emerged as a relatively more accepted method of contraception, lack of accurate information or misinformation regarding temporary methods, as well as limited access and affordability of modern temporary methods, contribute to the preference for female sterilization among women [15]. Empowering women with informed choices strengthens their confidence and commitment to using contraceptives [17]. Additionally, offering proper counselling with clear and accessible guidance upholds their right to information and supports their reproductive autonomy [18]. This paper outlines the usage of contraceptive methods among the married women aged 15–49 years in India, including the factors influencing contraceptive use.

## Data and methods

### Study setting and the data

The data for this study was derived from the fifth round of the National Family Health Survey (NFHS-5), conducted between 2019 and 2021 in India. The NFHS, which has similar structure as Demographic and Health Survey (DHS), is a periodic, cross-sectional, and multistage household survey designed to provide comprehensive data on population, health, HIV, and nutrition. Similar to the DHS program, which has conducted over 400 surveys in 90 countries, NFHS captures diverse dimensions of health and demographic data. The survey’s protocol, including the questionnaire, was approved by the Institutional Review Board of the International Institute for Population Sciences (IIPS) and ICF and was also reviewed by the US Centers for Disease Control and Prevention (CDC) [19].

For NFHS-5, a multistage sampling strategy was employed. In the first stage, primary sampling units (PSUs) were selected using probability proportional to size, followed by mapping and enumeration of households within the chosen PSUs. In the second stage, systematic sampling was used to select households for participation. The survey targeted all women aged 15–49 years who resided in the sampled households on the night preceding the interview. Ultimately, data from 724115 women were collected, addressing topics such as family planning, socioeconomic conditions, and health at multiple levels. Of these, the final sample analysed for the study included 512408 women who were in a marital union at the time of the survey.

### Outcome variables

Our primary focus was on the utilization of different contraceptive methods among the married women aged 15–49 years. The age group of 15–49 years has been selected for this study as it represents the reproductive age range commonly used in demographic and health research, including contraceptive studies. Additionally, the National Family Health Survey (NFHS) reports data exclusively for this age group [19]. The contraceptive methods were classified into four groups: permanent methods (including male and female sterilization), long-acting reversible (LAR) methods (only LAR option available in India is intrauterine devices or "IUDs" ), short-term modern methods (such as male and female condoms, pills, foam and jelly, injectables, and diaphragm), and traditional methods (encompassing withdrawal, rhythm, and other traditional methods reported by the women). Women who did not adopt any method were categorized under the heading "no method". The categorization of contraceptive methods aligns with global and national frameworks, ensuring methodological consistency in analysing effectiveness, duration, and user reliance.

### Explanatory variables

We selected specific variables for analysis based on their potential impact and observed connections with the usage of various contraceptive methods. The independent variables used to evaluate the utilization of injectable contraceptive methods include socio-demographic factors, behavioural characteristics, and family attributes. Covariates encompass age, categorized into five groups: 15–19 years, 20–24 years, 25–29 years, 30–34 years, and 35 years and above. Additionally, we considered women's educational attainment, categorized as primary, secondary, and higher education levels. Other factors taken into account include caste (Scheduled caste, scheduled tribe, OBC, and others), religion (Hindu, Muslim, Christian, and others), wealth index (poor, middle, and high) (In NFHS, data on ownership of assets (e.g., television, car, refrigerator), housing characteristics (e.g., flooring material, toilet facilities, source of drinking water), and other durable goods are collected; using PCA, these variables are transformed into a single wealth score, which is then categorized into quintiles), place of residence (rural and urban), region, reason for discontinuing the last method, marital status, reason for discontinuing the last contraceptive method, and future plan for children. Exposure to mass media is also considered to assess knowledge regarding available contraceptive methods, with three variables: frequency of reading newspapers or magazines, frequency of listening to the radio, and frequency of watching TV. These variables are further divided into three responses: "not at all," "less than once a week," and "at least once a week." Those who responded"not at all"were categorized as having no mass media exposure, while others were categorized as having exposure.

### Analytical plan

To assess contraceptive use from earlier NFHS rounds and current method in use, cross-tabulation was performed, focusing on women in marital unions. The state-wise prevalence of contraceptive use was examined (Fig. 3), again categorizing contraceptive methods based on their effectiveness. Methods were categorized into two groups: more effective methods, including short-term modern methods, IUDs, and permanent methods, and less effective methods, comprising traditional approaches of contraception (Fig. 4).

Current utilization of different methods of contraception across different sociodemographic characteristics was estimated using the weighted prevalence. To account for the factors associated with utilization of different method of contraception logistic regression was carried out for all the independent variables with the outcome variable. Adjusted odds ratios (ORs) were presented with 95% CI. The entire analysis was performed using the STATA 17.0 statistical software. Sampling weights provided in the dataset were applied in all analyses to account for the survey design and ensure representativeness. The analyses were performed using Stata 17 statistical software [20]. As NFHS-5 data is publicly available, ethical clearance was not necessary for this study.

## Results

**Fig. 1:**
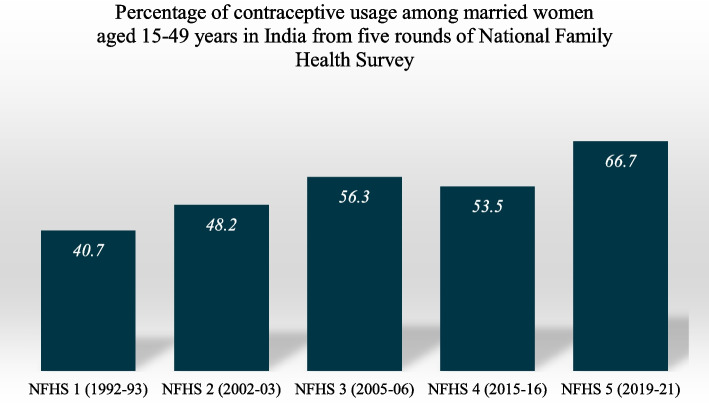
Percentage of contraceptive usage among married women aged 15–49 years in India from five rounds of National Family Health Survey

Contraceptive use among married women aged 15–49 years has seen a significant increase over the years, rising from 40.7% in NFHS-1 (1992–1993) to 66.7% in NFHS-5 (2019–2021). However, there was a slight decline observed in NFHS-4 (2015–2016), where usage dropped to 53.5% from 56.3% reported in NFHS-3 (2005–2006) (Fig. 1).

**Fig. 2:**
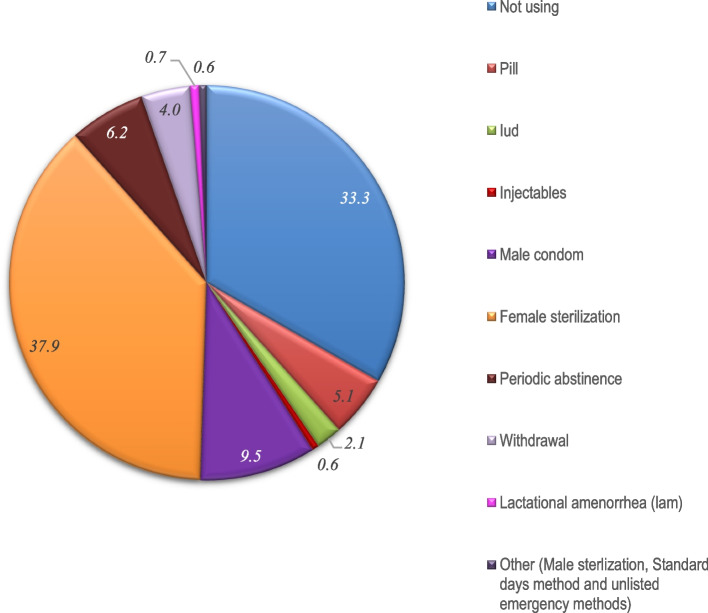
Contraceptive methods used by the married women aged 15–49 years in India (NFHS-5, 2019–21)

Regarding current contraceptive method, 33.3% of the surveyed women reported not using any form of contraception (Fig. 2). Among those who did, permanent contraceptive methods was the most prevalent, with 37.9% of married women opting for female sterilization. Usage of long-acting reversible contraceptives, such as intrauterine devices (IUDs), remained low at only 2%. Additionally, oral contraceptive pills were used by 5% of women, while 9.5% women reported their method as condoms utilized by their partners. Traditional methods also accounted for a significant portion of contraceptive practices, with 6.2% of women choosing periodic abstinence and 4% relying on the withdrawal method (Fig. 2).

**Fig. 3:**
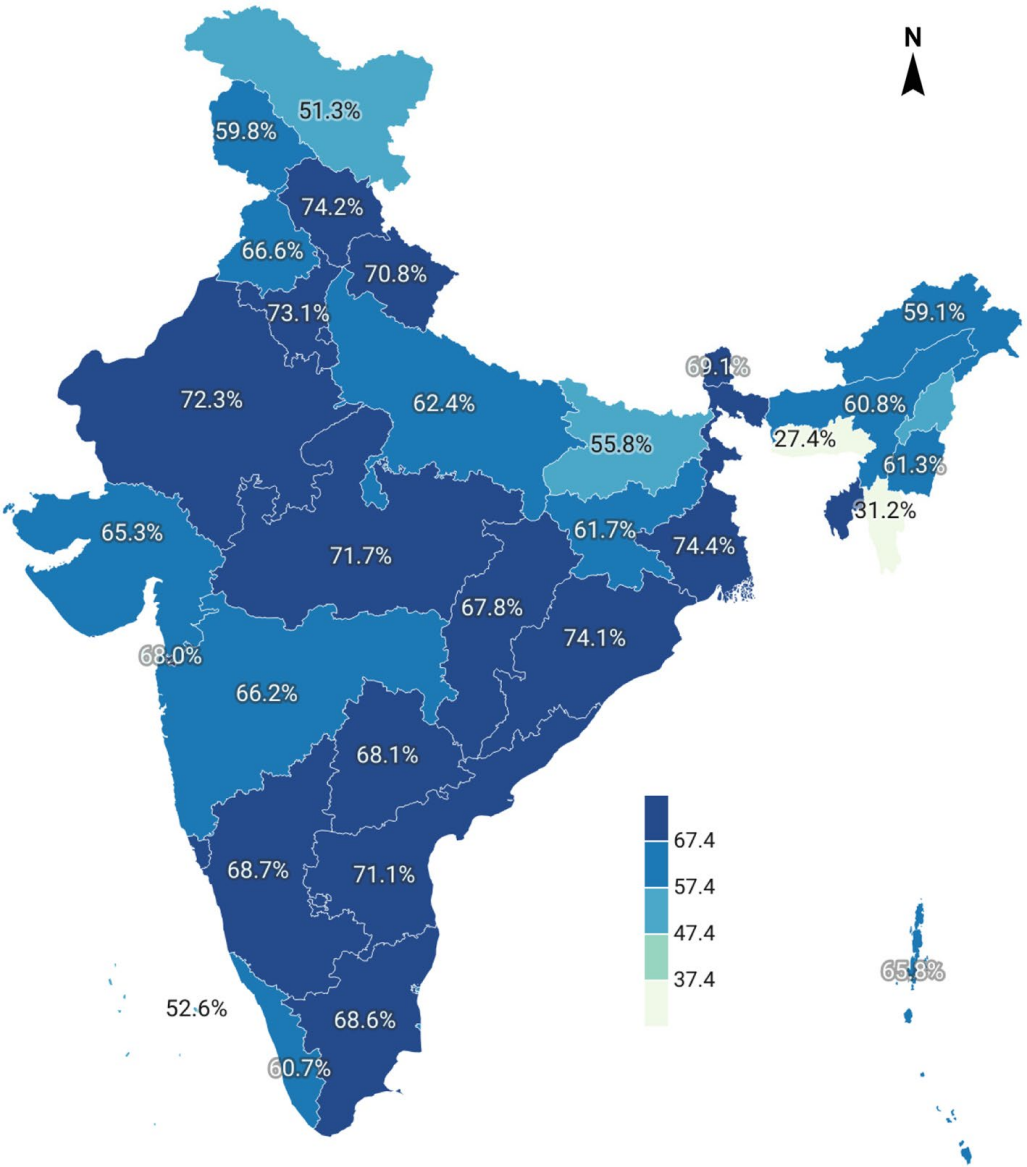
Map of India showing the percentage of use of contraceptive among the married women aged 15–49 years across the states of India, 2019–21. (Map not to scale). Source: Authors generated the map [21]

**Fig. 4:**
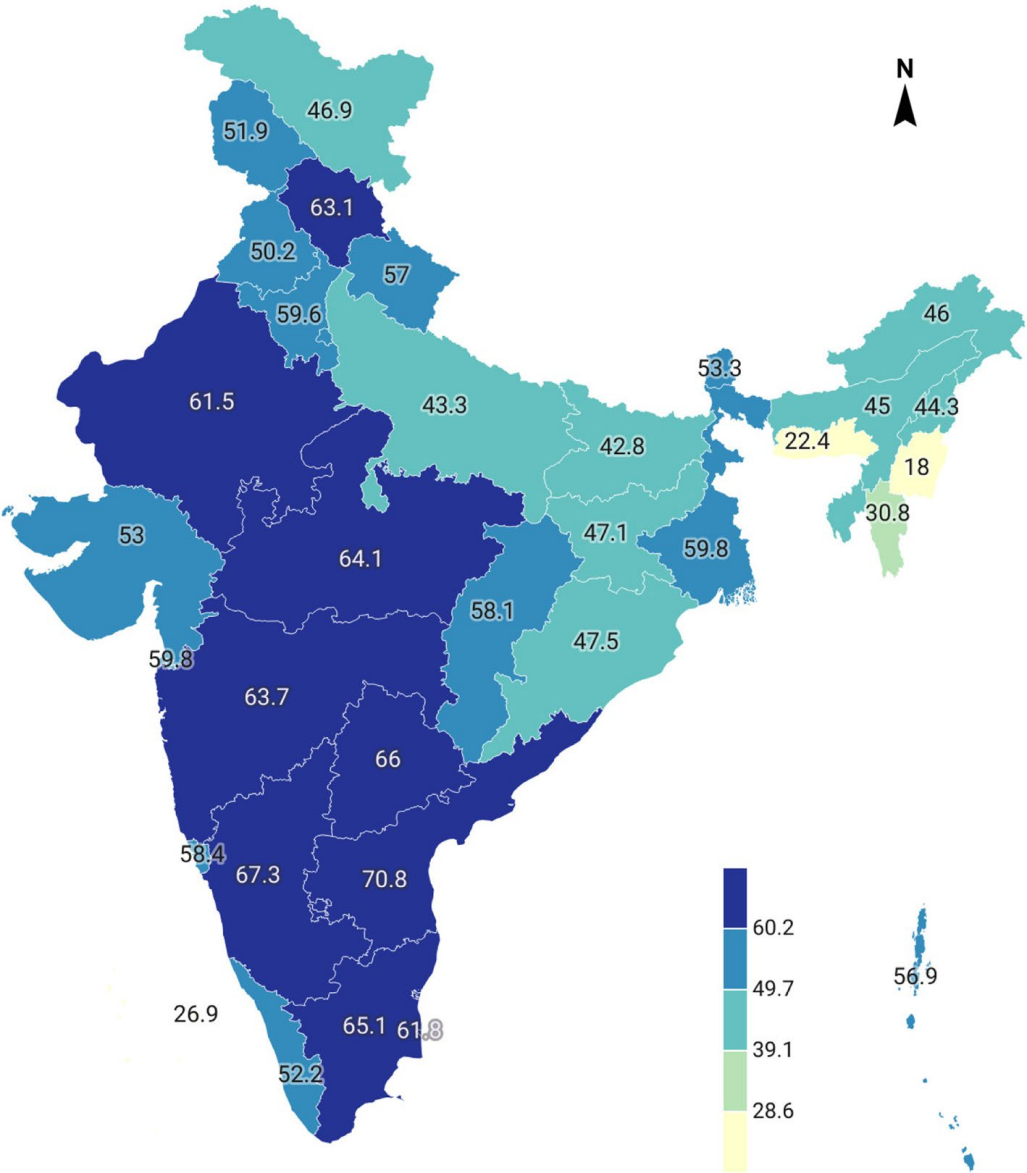
Map of India showing the percentage of use of effective method of contraceptive (methods other than traditional methods) among the married women aged 15–49 years across the states of India, 2019–21. (Map not to scale). Source: Authors generated the map [21]

In several Indian states, more than 70% of women reported using some form of contraception. These states include West Bengal, Himachal Pradesh, Odisha, Haryana, Rajasthan, Madhya Pradesh, Andhra Pradesh, and Uttarakhand (Fig. 3). However, when contraceptive use was assessed by the effectiveness of methods, only Andhra Pradesh stood out, with more than 70% of women using effective methods, such as permanent, short term modern methods, or LAR methods, as opposed to traditional methods like withdrawal or periodic abstinence (Fig. 4).

Women aged 15–24 years have the highest proportion of non-users (60%), but as age increases, permanent method adoption becomes prominent, with 54.9% of women aged 35 + relying on such methods. While 48.9% of uneducated women rely on permanent methods, women with higher education prefer short-term modern methods (24.1%) and IUDs (3.6%) (Table 1). Scheduled Castes and Tribes show higher reliance on permanent methods (around 40%), whereas “Others” caste groups have higher usage of traditional methods (12.2%). Among religions, Hindu women have higher adoption of permanent methods (40.9%), while Muslim women prefer short-term modern methods (22.5%). Wealth quintile shows a socioeconomic gradient, where richer women exhibit higher use of short-term modern methods (17.9%), in contrast to poorer women, among whom non-users are more prevalent (35.8%). South India leads in the adoption of permanent methods (59.6%), while North-East India shows the highest use of short-term methods (30%). In contrast, East and Central regions report relatively higher reliance on traditional methods. Urban women showing greater use of IUDs (2.7%) and short-term modern methods (18.5%) compared to rural women. Women with two or more children show higher use of permanent methods (51.7%), whereas non-users dominate among those with no children (83.2%) (Table 1).

**Table 1:** Percent distribution of current contraceptive method among currently married women aged 15–49 years by background characteristics, India, 2019–21

Background characteristics	Non user	Permanent methods	LAR methods (IUDs)	Short-term modern methods	Traditional methods	Total
Age						
15–24	60.0	6.6	2.9	18.0	12.5	85,499
25–34	33.0	32.0	2.8	19.7	12.4	1,92,837
35 +	23.7	54.9	1.2	10.4	9.7	2,34,072
Educational attainment						
No education	29.9	48.9	0.9	9.5	10.8	1,40,434
Primary	28.6	46.4	1.4	13.2	10.4	70,882
Secondary	34.6	34.6	2.6	16.7	11.4	2,35,169
Higher	40.8	19.5	3.6	24.1	12.1	65,923
Caste						
Scheduled caste	33.0	40.1	1.9	14.0	11.0	1,10,678
Scheduled tribe	35.6	41.0	2.3	10.5	10.6	47,031
OBC	33.6	40.7	2.0	13.0	10.8	2,20,828
Others	32.2	31.6	2.4	21.5	12.2	1,33,871
Religion						
Hindu	32.1	40.9	2.1	14.1	10.8	4,19,787
Muslim	39.8	21.9	2.0	22.5	13.8	67,453
Christian	38.2	42.2	3.5	8.1	8.0	11,195
Others	32.8	32.4	3.2	19.7	11.8	13,973
Exposure to mass media						
No	37.7	35.3	1.4	12.9	12.8	1,25,041
Yes	31.9	39.2	2.4	15.9	10.7	3,87,367
Wealth quintile						
Poor	35.8	36.8	1.6	13.5	12.3	1,98,631
Middle	32.3	42.6	1.9	12.9	10.2	1,04,661
Rich	31.4	37.3	2.7	17.9	10.6	2,09,117
Place of residence						
Urban	30.7	36.6	2.7	18.5	11.5	1,60,591
Rural	34.5	39.0	1.8	13.7	11.1	3,51,817
Region						
North	28.8	33.5	3.0	21.7	13.1	70,368
Central	34.8	28.7	1.5	19.6	15.4	1,21,122
East	34.0	32.2	1.7	16.5	15.7	1,22,125
North east	40.7	9.2	3.4	30.0	16.7	18,816
West	34.1	44.9	2.3	12.9	5.8	73,492
South	31.9	59.6	2.4	3.3	2.8	1,06,486
Living Children						
No children	83.2	0.6	0.2	10.7	5.3	49,449
Single child	48.3	8.4	4.1	23.6	15.6	1,01,548
Two or more children	22.2	51.7	1.8	13.4	10.8	3,61,412
Among the living children if there is a son						
No	58.1	12.4	2.1	16.7	10.7	1,32,577
Yes	24.6	47.2	2.1	14.7	11.4	3,79,832
Desire for children^a^						
Planning within 2 years	73.1		1.7	15.2	9.9	62,080
Planning after two years	51.6		4.7	26.3	17.3	55,496
Undecided	60.9		3.1	22.0	14.1	20,771
Don't want	42.1		4.0	30.3	23.6	1,71,704
Husband's desire for children^a^						
Agrees with the woman	51.2		3.6	26.1	19.1	2,76,162
Husband disagreed	57.6		3.3	22.7	16.5	34,533
Total	33.3	38.2	2.1	15.2	11.2	5,12,408

^a^Not additive to the total because of missing cases

Compared to younger women (aged 15–24 years), women aged 25–29 years are more likely to adopt contraception (OR = 1.16) (Table 2). The likelihood increases further for women aged 30–34 years, (odds for using contraception rise to 1.46 for non-users vs. users and 1.32 for effective method users). Educated women are significantly more likely to use effective methods (OR = 1.26, 1.43 and 1.73 for primary, secondary and higher education respectively). Women with higher education are also more likely to adopt specific methods, such as IUDs (OR = 1.84) and short-term modern methods (OR = 1.8). Compared to Scheduled Caste women, Scheduled Tribe women show lower odds of using effective methods (OR = 0.92) and short-term methods (OR = 0.82) but have higher odds for IUD use (OR = 1.62). Muslim women have higher odds of adopting effective methods (OR = 1.17) as compared to their Hindu counterparts but lower odds of IUD use (OR = 0.79). Conversely, Christian women show lower odds of using any method except for IUD use (Table 2).

**Table 2:** Factors associated with methods of contraception among the married women aged 15–49 years, India (NFHS-5, 2019–21)

Background characteristics	Adjusted odds ratio (95% CI)			
**Age**	**Non user Vs user**	**Non user vs effective method user**	**Non user Vs IUD**	**Non user Vs short term modern method**
15–24 ®				
25–29	1.16***(1.13–1.19)	1.15***(1.12–1.18)	1.17***(1.11–1.24)	1.21***(1.17–1.24)
30–34	1.46***(1.42–1.5)	1.32***(1.28–1.36)	1.43***(1.34–1.52)	1.53***(1.48–1.58)
35 +	1.11***(1.08–1.14)	0.92***(0.89–0.94)	0.99 (0.93–1.06)	1.01 (0.98–1.05)
Educational attainment				
No education ®				
Primary	1.18***(1.15–1.21)	1.26***(1.22–1.3)	1.35***(1.26–1.45)	1.28***(1.23–1.32)
Secondary	1.35***(1.32–1.38)	1.43***(1.39–1.46)	1.8***(1.69–1.91)	1.45***(1.41–1.49)
Higher	1.55***(1.5–1.6)	1.73***(1.68–1.79)	1.84***(1.7–1.99)	1.8***(1.73–1.86)
Caste				
Scheduled caste ®				
Scheduled tribe	0.95**(0.92–0.98)	0.92***(0.89–0.95)	1.62***(1.51–1.73)	0.82***(0.79–0.85)
OBC	1.03**(1.01–1.05)	0.94***(0.92–0.97)	1.13***(1.07–1.2)	0.96**(0.94–0.99)
Others	1.09***(1.07–1.12)	1.11***(1.08–1.14)	1.2***(1.13–1.28)	1.13***(1.09–1.16)
Religion				
Hindu ®				
Muslim	0.95***(0.92–0.97)	1.17***(1.14–1.2)	0.79***(0.74–0.84)	1.09***(1.06–1.12)
Christian	0.62***(0.6–0.65)	0.74***(0.71–0.77)	1.29***(1.2–1.39)	0.48***(0.45–0.5)
Others	0.94***(0.9–0.97)	1.01 (0.97–1.05)	1.22***(1.13–1.32)	0.93**(0.89–0.97)
Exposure to mass media				
No ®				
Yes	1.28***(1.26–1.31)	1.26***(1.24–1.29)	1.38***(1.31–1.46)	1.35***(1.32–1.39)
Wealth quintile				
Poor ®				
Middle	1.05***(1.02–1.07)	1.06***(1.04–1.09)	1.02 (0.96–1.07)	1.09***(1.06–1.12)
Rich	1.19***(1.16–1.22)	1.22***(1.19–1.26)	1.12***(1.06–1.19)	1.29***(1.25–1.33)
Place of residence				
Urban ®				
Rural	0.82***(0.8–0.84)	0.86***(0.84–0.88)	0.8***(0.76–0.84)	0.81***(0.79–0.83)
Region				
North ®				
Central	0.87***(0.85–0.89)	0.8***(0.78–0.82)	0.44***(0.41–0.47)	0.85***(0.82–0.87)
East	0.94***(0.91–0.96)	0.63***(0.61–0.65)	0.53***(0.5–0.57)	0.73***(0.71–0.76)
North east	1.25***(1.21–1.28)	1.21***(1.18–1.25)	0.87***(0.82–0.94)	1.35***(1.31–1.4)
West	0.54***(0.53–0.56)	0.71***(0.69–0.73)	0.63***(0.58–0.67)	0.55***(0.53–0.57)
South	0.24***(0.23–0.25)	0.33***(0.32–0.34)	0.69***(0.64–0.73)	0.17***(0.16–0.17)
Living Children				
No children ®				
Single child	3.42***(3.31–3.53)	2.69***(2.59–2.79)	26.01***(20.81–32.51)	2.86***(2.75–2.97)
Two or more children	4.47***(4.31–4.63)	3.23***(3.1–3.36)	37.34***(29.79–46.79)	3.66***(3.5–3.82)
Among the living children if there is a son				
No®				
Yes	1.23***(1.2–1.25)	1.16***(1.13–1.18)	1.32***(1.25–1.38)	1.23***(1.2–1.26)
Desire for children *				
Planning within 2 years®				
Planning after two years	1.79***(1.74–1.84)	1.57***(1.52–1.62)	2.65***(2.46–2.85)	1.73***(1.67–1.79)
Undecided	0.98 (0.95–1.02)	1.06**(1.02–1.1)	1.33***(1.22–1.45)	1 (0.96–1.05)
Don't want	1.6***(1.56–1.64)	1.44***(1.39–1.48)	2***(1.87–2.15)	1.6***(1.55–1.65)
Husband's desire for children *				
Agrees with the woman ®				
Husband disagreed	0.81***(0.79–0.83)	0.91***(0.89–0.94)	1.01 (0.96–1.07)	0.81***(0.79–0.84)
Constant	0.14***(0.13–0.15)	0.08***(0.08–0.09)	0***(0–0)	0.08***(0.08–0.09)

Significance level: * < 0.1, ** < 0.05 and *** < 0.001

®:Reference category

Women with exposure to mass media are significantly more likely to use any contraceptive method (OR = 1.28) and effective methods (OR = 1.26) compared to those with no exposure. Women from rich and middle wealth quintile have significantly higher odds of contraceptive use compared to poor women, with the richest group showing the strongest association (OR = 1.22 for effective methods and OR = 1.29 for short-term modern methods). Women residing in rural areas exhibit lower odds of using contraceptives also that of using effective methods (OR = 0.82, 0.86 respectively) compared to their urban counterpart. Women in the North-East have higher odds of using short-term modern methods (OR = 1.35), whereas women in the South have substantially lower odds for all methods (Table 2). Women who have mothered any child have exponentially higher odds of adopting contraceptive methods compared to women without children. Women who plan to have children after two years show higher odds of adopting effective methods (OR = 1.57) and IUDs (OR = 2.65). Women who no longer desire children are more likely to adopt effective methods (OR = 1.44). However, discordance in fertility desires with husbands reduces contraceptive use; women whose husbands disagreed on fertility preferences had lower odds of adopting methods (OR ~ 0.81).

## Discussion

The acceptance and utilization of contraceptive methods among married women in India are influenced by multiple factors operating at individual, family, and community levels. This study provides valuable insights into contraceptive usage patterns and associated determinants among women, revealing significant gaps in the adoption of effective and long-term methods. The findings highlight that the use of long-term reversible contraceptive methods, such as IUDs, remains notably low across all demographic groups. A significant proportion of women continue to rely on traditional methods or use no method at all, even after achieving their desired family size. This underutilization may be attributed to the challenges of accessing healthcare facilities or the need for professional administration of methods like IUDs, which are perceived as inconvenient. Women who use short-term modern methods often make this decision after concluding their reproductive plans, which is encouraging given the traditional dominance of male or elder family authority over such decisions in many Indian households.

Age emerges as a key factor influencing contraceptive choices. Women aged 35 + years show a higher preference for permanent methods, particularly sterilization. However, the adoption of sterilization remains lower among younger women aged 15–24 years (6.6%). This lower rate among younger women aligns with findings from district-level surveys and reflects the evolving, less concrete reproductive decisions of younger women compared to older, more experienced counterparts [22]. Educational attainment significantly shapes contraceptive behaviour. Women with higher education levels demonstrate greater acceptance of long-term reversible methods and short-term modern methods, consistent with global patterns observed in other countries, such as the Democratic People’s Republic of Korea and Zimbabwe [23, 24]. However, among the women belonging to the general caste group which was categorized as “other”, traditional methods remain prevalent, with 12% relying on such methods. The findings further suggest that cultural and structural barriers may deter the adoption of effective contraceptive methods, necessitating interventions to address these challenges.

Religious beliefs also influence contraceptive choices. Christian women show lower adoption of short-term modern methods, while their reliance on permanent methods is considerably high. This could be attributed to both individual choices and religious beliefs regarding contraception. Similarly, the higher usage of contraceptives among Muslim women highlights an emerging trend of increasing awareness and acceptance of modern methods. Exposure to mass media significantly enhances contraceptive adoption across all method categories. Women exposed to mass media are likely to have greater awareness of the available contraceptive options and improved understanding of reproductive health choices [25]. Wealth status further exacerbates disparities in contraceptive use, with women in higher wealth quintiles showing higher uptake of modern contraceptives. This trend reflects the greater availability of financial resources and better access to healthcare facilities among wealthier women. Regional variations in contraceptive behaviour are also noteworthy. Women in the north-eastern region display distinct patterns of contraceptive use, with higher adoption of short-term modern methods, possibly reflecting diverse cultural practices and attitudes toward family planning. By contrast, the southern region reports a higher preference for permanent methods, such as sterilization. These findings underscore the need for region-specific family planning programs to address diverse cultural and socioeconomic dynamics.

Factors such as healthcare access, cultural influences, and misinformation play a critical role in shaping contraceptive use among women. Limited access to healthcare facilities, particularly in rural and underserved regions, restricts the availability of contraceptive methods and counselling services, deterring women from adopting effective options. Cultural norms and societal pressures often discourage contraception, especially in communities where early childbearing and larger family sizes are traditionally valued. Additionally, misinformation and myth**s** surrounding contraception, such as perceived side effects or fertility concerns, contribute to hesitancy and fear among women. Addressing these barriers through improved healthcare infrastructure, culturally sensitive education programs, and accurate information dissemination is essential for enhancing contraceptive adoption and empowering women to make informed reproductive choices.

The study also identifies a strong association between fertility intentions and contraceptive use. Women uncertain about their next reproductive decision or those planning to delay pregnancies are more likely to use no contraceptive method, reflecting their indecision and lack of clarity. Furthermore, the analysis shows that women whose family planning preferences are not aligned with their husbands are significantly less likely to use any contraceptive method. This finding underscores the importance of spousal support in reproductive decision-making, as disagreements may limit women’s access to healthcare services and related resources. The uptake of permanent methods and long-acting reversible contraceptive (LARC) methods remains exceptionally low in India, despite evidence of their effectiveness in achieving desired inter-pregnancy intervals and reducing unintended pregnancies. Women’s reluctance to adopt these methods may stem from underestimating pregnancy risks, plans for abstinence, or provider-specific barriers such as limited availability of supplies and counselling at healthcare facilities. Although there has been an increase in IUD usage over time, this method remains underutilized. Ensuring the availability of contraceptives and addressing provider-side challenges at health facilities, particularly during pregnancy-related visits, can encourage adoption and prevent discontinuation.

The preference for traditional methods over effective modern methods remains another significant concern. This reliance increases the risk of inconsistent contraceptive use and unintended pregnancies. To enhance reproductive health outcomes, it is imperative to reduce barriers to contraceptive adoption by ensuring prompt availability and accessibility of effective methods. Policies and programs must prioritize outreach to women in need of contraception, particularly those from disadvantaged communities.

This study’s strength lies in its use of a nationally representative dataset, which offers comprehensive insights into contraceptive behaviour among currently married women in India. However, certain limitations must be acknowledged. The data originate from a cross-sectional survey, which may be subject to recall bias and limits causal interpretations.

## Conclusion

The findings highlight that the majority of currently married women in India continue to either use no contraceptive method or rely on less effective traditional methods. This underscores the critical need to strengthen contraceptive services by addressing structural barriers and enhancing awareness about effective methods. India’s Family Planning 2030 initiative, launched in 2022, emphasizes the importance of timing and spacing of pregnancies to improve maternal and child health outcomes.

The success of India’s family planning program depends significantly on the collaborative efforts of policymakers, researchers, service providers, and users. To address the persisting gaps, there is an urgent need to intensify existing family planning programs, with a particular focus on married women. Targeted interventions must emphasize the risks associated with early childbearing, gap between childbirths, while equipping women with the knowledge, skills, and resources to delay or take necessary gap between pregnancies if they so desire. Furthermore, the study’s findings reinforce the need to strengthen contraceptive services to improve access and adoption of effective methods, particularly in remote regions and among vulnerable groups. Enhancing awareness through education and mass media campaigns, ensuring equitable service delivery with addressing cultural and structural barriers will be critical to achieving the goals of India’s Family Planning 2030 initiative**.**

## Data Availability

No datasets were generated or analysed during the current study.
